# A feasibility study to assess the validity of administrative data sources and self-reported information of breast cancer survivors

**DOI:** 10.1186/s13584-016-0111-6

**Published:** 2016-12-01

**Authors:** Rola Hamood, Hatem Hamood, Ilya Merhasin, Lital Keinan-Boker

**Affiliations:** 1 Leumit Health Services, Haharoshet 17, Karmiel, Israel; 2 School of Public Health, University of Haifa, Haifa, Israel; 3 Leumit Health Services, Netanya, Israel; 4 Ministry of Health, Israel Center for Disease Control, Ramat Gan, Israel

**Keywords:** Breast cancer, Validity, Agreement, Administrative data, Self-report, Medical record

## Abstract

**Background:**

Cancer survivorship has increasingly become the focus of research due to progress in early detection and advancements in the therapeutic approach, but high-quality information sources for outcomes, potential confounders and personal characteristics present a challenge. Few studies have collected breast cancer care data from mixed data sources and validated them, and to the best of our knowledge, none so far have been conducted in Israel, where National Health Insurance Law assures universal health care, delivered through four health care funds with computerized administrative, pharmaceutical and medical databases.

This validation study is aimed to assess the accuracy and completeness of information on cancer care and health outcomes using several research tools, before embarking on a full-scale study aimed to evaluate the long-term treatment-related health adverse outcomes in a cohort of breast cancer survivors.

**Methods:**

One hundred twenty randomly sampled female patients diagnosed with primary breast cancer in years 2000–2010 in northern Israel, who are members of the “Leumit” healthcare fund, were included. Data sources included “Leumit” medical records, the National Cancer Registry and a self-report questionnaire. The questionnaire was completed by 99 % of the women contacted. The accuracy of the information regarding cancer care was assessed with the reference standard set as one of the research tools, varying per the characteristic being under investigation. For example: health outcomes and medical history were validated against “Leumit” medical records, while construct validity of the self-reported questionnaire served to assess the prevalence of chronic pain. Agreement, predictive values, correlations, and internal consistency were calculated. Logistic regression models were constructed to assess potential predictors of correct responses.

**Results:**

The overall level of agreement (*Kappa*) was almost perfect for demographics and outcomes, above 0.8 for treatments and chronic pain, while only fair to moderate for most of the self-reported medical history. Correct responses of medical history were associated with Jewish ethnicity, recency of breast cancer diagnosis, and family history of cardiovascular disease. The internal consistency of the quality-of-life scale was above 0.9.

**Conclusion:**

In the absence of a national registry for cancer care, a mixed methodology for data collection is the most complete source.

**Trial registration:**

Trial registration number Not available. This is an observational study with prospective data collection and no intervention; therefore, trial registration number is not required.

## Background

Cancer survivorship has increasingly become the focus of research due to progress in early detection and advancements in the therapeutic approach. The goal of survivorship research is to understand, and thereby reduce, the adverse outcomes of cancer diagnosis and treatment [1]. Breast cancer survivors may develop late treatment-induced organ damage and functional disabilities, and many of them are likely to suffer from unendurable chronic pain which might irreversibly alter the quality of their lives. To effectively conduct a survivorship study, one is in need of information on potential adverse outcomes, explanatory variables, such as cancer clinical characteristics and primary treatment, as well as potential confounders, including sociodemographic factors, lifestyle and health behavior. Good and valid information on all of these variables is, in fact, a challenge.

Medical records have frequently been considered the “gold standard” means for retrieving detailed breast cancer clinical information; however, this method of data collection, especially if not fully digitalized, can be expensive, time-consuming and labor intensive [2–4]. In contrast, administrative claim databases, are easier to obtain, but are valid only when payment is not bundled [4] and covered services have specific codes for reimbursement. However, both medical records and administrative claims were not primarily intended to be used for research or surveillance purposes, and accordingly their quality of data is oftentimes questioned [5].

Cancer registries, on the other hand, have been designed specifically for epidemiological research and cancer control. Completeness of breast cancer case-finding has been shown to be very high, yet the validity and completeness of cancer registry care data have not been well-established [6].

Self-report questionnaires have been adopted as a more feasible option for obtaining information on cancer treatment [2, 7] but the appraisal of cancer long-term impact on the quality of life of breast cancer survivors has not been commonly prioritized in these surveys.

Linking these diverse but complementary data sources can provide unique cancer care information, provided the collected data are complete and valid [8, 9]. To date, few studies have invested time and effort in gathering comprehensive breast cancer care data from mixed sources [10], and even fewer have endeavored to validate these data so to avoid disseminating erroneous information in the clinical setting [2, 3]. To the best of our knowledge, none of these validation studies were carried out in Israel, where National Health Insurance Law assures universal healthcare, delivered through four healthcare funds.

The objective of the present feasibility study was to assess the validity of several research tools in reporting information on breast cancer care and certain health outcomes and to identify potential sources of bias in outcome estimation [11] before embarking on a full-scale study intended to evaluate the long-term treatment health-related adverse outcomes in a cohort of breast cancer survivors.

## Methods

### Study population

Subjects of this feasibility study were female members of Leumit Health Services (LHS), a nonprofit Israeli health maintenance organization (HMO). All enrolled participants were breast cancer patients, at least one-year survivors, who were treated for early-stage or regionally advanced invasive breast cancer between January 1, 2000 and December 31, 2010. The upper limit of study entry was set to 2010 to guarantee that the follow-up period was extended sufficiently (at least five years) to capture adverse outcomes that are associated with long latency periods.

Patient medical record stored at LHS was used to identify patients with a diagnosis of first primary breast cancer (classified according to the *International Classification of diseases, Ninth Revision (ICD-9*) codes: 174 (174.0-174.9)). An approximate 20 % random sample of all women diagnosed with breast cancer and treated in Rambam Medical Center, a healthcare tertiary hospital located in the Israeli northern district, was selected using a sampling strategy stratified by year of diagnosis. The rationale for restricting our study population to a single institution was twofold: First, inasmuch as the objective of the full-scale study is to evaluate cancer treatment impact on health of breast cancer survivors, the element of variance in distribution of treatments among study population is of central importance. Rambam Medical Center, which is the only institution in the north of Israel that offers combined treatment for breast cancer according to a standard protocol, was therefore an ideal choice. Secondly, we intended to estimate the degree of Hebrew comprehension among the Arab participants, whose majority resides in the north of the country, as this language was used in the delivery of the questionnaire.

**Fig. 1:**
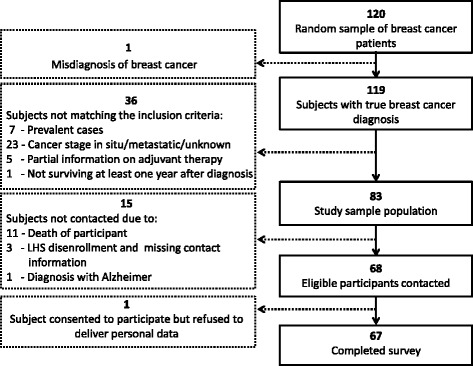
Survey sample flow chart

The initial eligibility screen identified 120 patients. By linkage with the Israel National Cancer Registry (INCR), 37 patients were excluded for not meeting the inclusion criteria. Of the remaining 83 eligible candidates for the administration of a constructed questionnaire, 15 subjects were not contacted due to death, LHS disenrollment and missing contact information, or degenerative mental illness, leaving a total 68 women who were contacted. 67 subjects completed the survey (Fig. 1).

### Data collection

#### Self-report questionnaire

A 77-item questionnaire written in Hebrew was developed to capture information that is not routinely registered in administrative databases. The questionnaire comprised of six main domains: (1) demographics, (2) lifestyles (3) medical history, (4) reproductive history, (5) outcome assessment of chronic pain and related sequelae, including allodynia, paresthesia, phantom sensations and fatigue, and (6) quality of life assessment using the medical outcomes study 36-item short-form health survey (SF-36) [12].

Questions on demography, medical and reproductive history, and lifestyles were adapted from the Israeli National Health Interview Survey [13]. The questionnaire did not include items on cancer treatments to avoid raising the participant's awareness to the main study questions and thus to keep the respondent bias to a minimum. Additionally, we do not expect breast cancer survivors, especially those diagnosed in earlier years, to recall information pertaining to their treatment.

To assess the appropriateness of the questionnaire, a two-stage pretest was performed. The first stage was an individually-based expert review. Three researchers from the academia and the clinical practice setting conducted independent reviews on the assigned questionnaire for the appraisal of potential problems with the data collection instrument, such as glitches in wording of questions, or need for additional items. A revised version of the instrument was administered to participants of the current pilot study in the form of telephone interviews, except for one face-to-face interview. If pretesting indicated that there was a low likelihood of obtaining sufficiently sound and consistent data, troublesome items were dropped or revised accordingly.

#### Medical record abstraction from leumit health services registries

LHS patient data are collected in real time from all levels of care and automatically stored in the patient electronic medical record, then downloaded on a daily basis to LHS central databases. This registry has been described elsewhere [14]. We used standard data collection forms to abstract detailed patient information on demographics, breast cancer characterization and treatments, medical history, medications and supplements, and vital status. Follow-up information on potential outcomes included incidence of cardiovascular disease, osteoporosis, fractures and diabetes, occurring at least one year post breast cancer diagnosis.

#### Leumit administrative databases

Breast cancer care data were obtained from three administrative datasets of LHS: 1) demographic registry; 2) diagnoses and procedures billing database; and 3) pharmacy claims. The demographic registry includes basic demographic and geographic information. The billing dataset contains information on services, diagnoses, and procedure interventions encountered from ambulatory care (same-day surgery, day procedures, emergency department visits, and community rehabilitation program services) or inpatient settings, coded using the ICD-9 coding system. The pharmacy dataset includes patient and prescriber information plus drug and dosing details (days of supply, quantity, drug name, and medication class), coded using the anatomical therapeutic chemical classification system.

#### Abstraction from Israel National Cancer Registry

INCR is a statutory body in the Israel Ministry of Health responsible for collecting data on malignant disease incidence since 1960. Cancer reporting by hospitals, pathology and cytology laboratories and other health care providers has been mandatory since 1982. INCR covers the entire Israeli population, approximating 8.3 million, and its database is currently complete as of the end of December 2013 [15]. Demographic and clinical data collected from INCR included: date of birth, ethnicity and religion, breast cancer diagnosis date, laterality, clinical stage, and treatments.

#### Validation procedure

The administrative databases (LHS and INCR) were used to abstract data on all 119 subjects with breast cancer, whereas the self-report questionnaire contributed information on 67 respondents (see Fig. 1). Comparisons were confined to shared patients between research tools, and only patient characteristics shared by more than one data source were examined for accuracy and completeness. The dataset adopted as the reference standard varied as a function of the characteristic being under investigation. As for residence, ethnicity and cohabitation status, the directly reported information through questionnaires was considered the reference standard by virtue of being the most up to the minute data source. INCR was used as a reference with respect to mortality, since this information is routinely routed to INCR via the Israel National Population Registry; the latter requires by law notification of a resident death occurring in Israel or abroad. Primacy of data was credited to INCR as well in relation to breast cancer diagnosis date, laterality, axillary node dissection, and type of surgery, as these data originated in the hospital are directly transferred to INCR. Generally, LHS administrative databases record such information, however, due to bundled payment in which hospitals are paid a “lump sum” per patient regardless of how many tests or treatments the patient receives [16], there may be at times a paucity of cancer treatment specific details in LHS claims. LHS claims were considered the reference standard for adjuvant therapy considering that LHS is billed specifically per exploited service, whether it were an administered drug or the delivery of radiation treatment. INCR may be lacking such information since hospitals tend to supplement data on initial course of treatment, mostly details of surgery that is considered the first-line treatment, while ambulatory services of adjuvant therapy that are undertaken in later stages are often underreported [6].

LHS individual medical records were reviewed for recovering missing information and the ascertainment of administrative outcome codes and reported medical history. The diagnostic criteria included discharge summaries, specialist notes, laboratory tests, referrals, signals and images, medical procedures and prescriptions. In addition, being an outcome study, it was essential to develop further validation algorithms of administrative diagnosis codes to guarantee minimal misclassification of outcome (Table 1). Chronic pain was not validated against medical charts; its validation was based instead on degree of concordance with the SF-36 self-reported measure of pain [17] (item 21: How much bodily pain have you had during the past 4 weeks?). The 6-response choices were dichotomized to: none to very mild, and mild to very severe.

**Table 1:** Validation algorithms of study health-related adverse outcomes

Outcome parameter (ICD-9 codes)^a^		Validation algorithm
Cardiovascular disease (410–414, 426–428, 430–438)	Ischemic heart disease (410–414)	• Evidence of angina pectoris symptoms or acute coronary syndrome, and/or • Imaging report (Angiography)
	Congestive heart failure (428)	• EF < 40 %, and/or • ≥3 dispensed prescriptions of diuretics, ACEI/ARB, or digoxin within six months following heart failure diagnosis [36]
	Conduction disorders and cardiac dysrhythmias (426–427)	• Direct-current cardioversion-defibrillation, pacemakers implantable cardioverter-defibrillators, radiofrequency ablation, and/or • ≥3 dispensed prescriptions of antiarrhythmic agents within 6 months following event
	Stroke (430–438)	• Hospitalization discharge sheet indicating a cerebral event, and/or • Imaging report (CT, PET, MRI), and/or • ≥3 dispensed prescriptions of anticoagulant agents within 6 months following event
Fractures (800–829)		• Imaging report (radiographs), and/or • Orthopedic referrals • Operations on the musculoskeletal system (ICD-9 codes 76–84) • Orthopedic aftercare (ICD-9 code V54)
Osteoporosis (733.0)		• Bone mineral density (T-score ≤ −2.5) [37], and/or • ≥1 dispensed prescriptions of bisphosphonates or estrogen agonist/antagonist (raloxifene) within 1 year following event
Diabetes (250)		• HbA1c level ≥6.5 %, or FPG ≥ 126 mg/dL, or 2-hour PG ≥200 mg/Dl [38], and/or • ≥3 dispensed prescriptions of insulin and oral diabetes medications within 6 months following diabetes diagnosis
Chronic pain^b^		• Concordance with the Hebrew validated Short Form 36 (SF-36) [17]

*Abbreviations*: *ICD-9* International Classification of diseases, Ninth Revision, *EF* ejection fraction, *ACEI* angiotensin converting enzyme inhibitor, *ARB* angiotensin receptor blocker, *CT* computerized tomography, *PET* positron emission tomography, *MRI* magnetic resonance imaging, *HbA1c* hemoglobin A1C, *FPG* fasting plasma glucose, *2-PG* 2-hour post glucose‑load plasma glucose

^a^ To account for possible variance in physician interpretation of ICD-9 codes and to increase the sensitivity of this tool, the current study referred only to the first three digits of ICD code for disease diagnosis [39], except for osteoporosis, which is a specific disorder of bone and cartilage

^b^ Chronic pain defined as pain persisting 3 months, beyond the normal time of healing [40]

### Statistical analysis

Agreement analysis was conducted with analysis restricted to parameters reported in more than one data source. For dichotomized variables, absolute agreement or proportion correct was first assessed. Next, *Kappa* statistic and 95 % confidence interval (CI) using the standard normal distribution [18] was used to evaluate inter-observer agreement. The interpretation of *Kappa* statistic was based on the suggested scale by Altman [19], which categorized the strength of agreement beyond chance as Poor (<0.20), Fair (0.21–0.40), Moderate (0.41–0.60), Good (0.61–0.80) and Very good (0.81–1.00). Where appropriate, we calculated positive and negative predictive values (PPVs and NPVs) and their 95 % CIs using the Wilson binomial method [20].

Pearson’s correlation coefficient (or Spearman’s rank correlation coefficient for ordinal variables) and 95 % CI were calculated based on Fisher’s r-to-z transformation to assess presence of linear relationship between continuous variables. Bland Altman plots [21] were constructed for quantifying inter-observer agreement. The Bland Altman method evaluates a bias (accuracy) between the mean differences of two quantitative measures, and an agreement interval (mean ± 1.96 standard deviation) within which 95 % of the differences fall (precision). Were the assumption of normal distribution of differences not satisfied (Graphical inspection of histogram, or *P < .05* in Shapiro-Wilk test for normality), the fold empirical cumulative distribution plot (mountain plot) was used instead. The center of the mountain plot shows the median bias between the measures, while its tails show the propensity for the new method to deviate significantly from the comparison method [22].

Questionnaire construct validity was estimated by the strength of correlation between self-reported chronic pain intensity and self-reported number of pain locations. Chronic pain intensity was rated on a 10-likert scale as 0 = no and 10 = unendurable pain, while number of pain locations ranged from 0 to 4 (area of the operated breast, armpit, arm, body side). Similarly, the validity of reporting on employment transition was estimated by assessing its correlation with self-reported income instability following breast cancer diagnosis/treatment. Employment transition or income instability was categorized as 1 = no change or stable, 2 = upgraded (from part-time to full-time job, or from not working to part/full-time job) or increased, and 3 = downgraded (from full-time to part-time job or not working/retired, or from part-time job to not working/retired) or decreased, respectively. The internal consistency of the SF-36 subscales was analyzed using Cronbach’s alpha with minimum acceptable value of alpha set to 0.7. *T*-test was performed to determine significant differences in quality of life scores between cancer survivors and the general population.

Completeness of data elements in administrative databases (LHS and INCR) or the self-report questionnaire was calculated as a proportion of total study subjects (*n* = 119) or total respondents (*n* = 67), respectively. Association between accuracy of self-reported medical history and potential explanatory self-reported variables, including age at time of survey, breast cancer diagnosis year, cohabitation status (married/unmarried), ethnicity (Jews/Arabs), immigration status (yes/no), parity, number of education years attained, family medical history of cardiovascular disease or diabetes (yes/no), body mass index (BMI), and lifestyle behaviors of physical activity, smoking and healthy diet (yes/no), was appraised using logistic regression with responses classified as correct or incorrect relative to LHS medical records. Odds ratios (ORs) and 95 % CIs were calculated. Due to the relatively small sample size, cohabitation status and lifestyle behavior variables were dichotomized: Unmarried category comprised of all women not married (single, separated, widowed, or divorced), former smoker was considered as nonsmoker, and the frequency of fruits and vegetables consumption originally rated on an ordinal scale was converted to a dichotomous variable with daily servings ≥1 regarded as eating healthy diet.

Statistical significance was defined as a two-tailed *P < 0.05*. All statistical analyses were performed using SAS 9.2 (SAS Institute Inc., Cary, NC, USA) and MedCalc 15.10.1 (MedCalc Software, Ostend, Belgium).

The Institutional Review Boards of LHS and University of Haifa approved the research protocol and all participants provided oral informed consent.

## Results

**Fig. 2:**
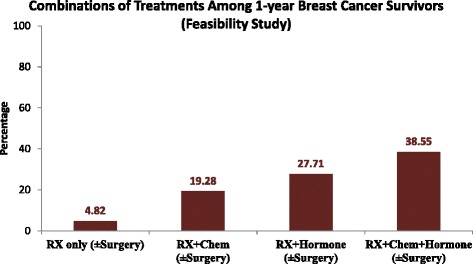
Distribution of treatments among 83 one-year breast cancer survivors. Notations: Rx, radiotherapy; Chem, chemotherapy; Hormone, hormone therapy. The proportions do not sum 100 % because more rare combinations of treatment (i.e., surgery only; etc.) are not presented

Of the 119 women identified from LHS databases as correctly having a diagnosis of breast cancer, 83 patients met the inclusion criteria. A dataset summarizing characteristics of the 83 survivors was created based on information synthesized from administrative databases, medical records, and the questionnaire (which contributed features of only alive women at time of survey) according to the aforementioned validation procedure. The mean age at diagnosis was 57 years for Jewish women and 50 years for Arab women (Table 2). Jewish women were more educated, less parous and reported chronic pain intensity to a lesser extent than their Arab counterparts. Distribution of combinations of treatments among these patients yielded four main mutually exclusive treatment categories that could be utilized in defining the exposure parameter in the full-scale study (Fig. 2). There were no significant differences in terms of breast cancer diagnosis year, age at diagnosis and treatments between the study population and the 67 interviewees (data not shown).

**Table 2:** Characteristics of the study population, by ethnicity

Characteristic	Breast cancer patients (*n* = 83, 100 %)^a^	
	Jewish women (*n* = 54, 65.1 %)	Arab women (*n* = 29, 34.9 %)
Demographics		
Age at breast cancer diagnosis		
≤ 39 years	2 (3.7)	8 (27.6)
40–49 years	13 (24.1)	8 (27.6)
50–59 years	21 (38.9)	8 (27.6)
≥ 60 years	18 (33.3)	5 (17.2)
Cohabitation status		
Married/living with a spouse	27 (50.0)	15 (51.7)
Unmarried^b^	18 (33.3)	7 (24.1)
Missing^c^	9 (16.7)	7 (24.1)
Education, mean^d^ (SD), year	13.3 (3.1)	7.2 (4.8)
Immigration status		
Yes (country birth not Israel)	34 (63.0)	1 (3.4)
No (country birth Israel)	11 (20.4)	21 (72.4)
Missing	9 (16.7)	7 (24.1)
Parity		
0	2 (3.7)	3 (10.3)
1	12 (22.2)	0 (0.0)
2	16 (29.6)	2 (6.9)
3	9 (16.7)	2 (6.9)
≥ 4	6 (11.1)	15 (51.7)
Missing	9 (16.7)	7 (24.1)
Breast cancer treatment and related effects		
Breast cancer diagnosis year		
2003–2005	21 (38.9)	8 (27.6)
2006–2008	18 (33.3)	12 (41.4)
2009–2010	15 (27.8)	9 (31.0)
Treatments		
Breast conserving	41 (75.9)	18 (62.1)
Mastectomy	12 (22.2)	10 (34.5)
Axillary lymph node dissection	32 (59.3)	21 (72.4)
Radiotherapy	48 (88.9)	27 (93.1)
Chemotherapy^e^	28 (51.9)	23 (79.3)
Hormone therapy^f^	41 (75.9)	20 (69.0)
Employment transition following diagnosis/treatment		
No change	25 (46.3)	17 (58.6)
Upgraded	1 (1.9)	0 (0.0)
Downgraded	19 (35.2)	5 (17.2)
Missing	9 (16.7)	7 (24.1)
Income change following diagnosis/treatment		
No change	26 (48.2)	17 (58.6)
Increased	0 (0.0)	1 (3.5)
Decreased	19 (35.2)	4 (13.8)
Missing	9 (16.7)	7 (24.1)
Weight change following diagnosis/treatment		
No change	15 (27.8)	8 (27.6)
Increased	16 (29.6)	11 (37.9)
Decreased	13 (24.1)	3 (10.3)
Don’t know	1 (1.9)	0 (0.0)
Missing	9 (16.7)	7 (24.1)
Chronic pain related to breast cancer		
Yes	31 (57.4)	19 (65.5)
No	14 (25.9)	3 (10.3)
Missing	9 (16.7)	7 (24.1)
Chronic pain intensity		
0 (no pain)	14 (25.9)	3 (10.3)
1–3 (light pain)	5 (9.3)	3 (10.3)
4–7 (moderate pain)	26 (48.2)	10 (34.5)
8–10 (severe pain)	0 (0.0)	6 (20.7)
Missing	9 (16.7)	7 (24.1)
Number of chronic pain locations		
0	14 (25.9)	3 (10.3)
1–2	24 (44.4)	9 (31.0)
3–4	7 (13.0)	10 (34.5)
Missing	9 (16.7)	7 (24.1)
Lifestyles		
Tobacco use		
Current smoker	7 (13.0)	2 (6.9)
Former smoker	7 (13.0)	1 (3.5)
Never smoker	31 (57.4)	19 (65.5)
Missing	9 (16.7)	7 (24.1)
Physical activity		
Yes	33 (61.1)	13 (44.8)
No	12 (22.2)	9 (31.0)
Missing	9 (16.7)	7 (24.1)
Daily servings of fruits and vegetables		
0	0 (0.0)	0 (0.0)
1–2	28 (51.9)	18 (62.1)
≥ 3	17 (31.5)	4 (13.8)
Missing	9 (16.7)	7 (24.1)
BMI, mean (SD), kg/m^2^	27.4 (4.9)	29.5 (6.3)

*Abbreviations*: *SD* standard deviation, *BMI* body mass index

^a^ 83 Eligible participants identified by both LHS and INCR records; of them 67 completed the survey questionnaire. Data are presented as No.(%) unless otherwise noted; percentages may not sum to 100 due to rounding

^b^ Unmarried category comprised of all women not married: divorced, separated, widowed, or single

^c^ Missing information refers to information elicited from questionnaires, since not all subjects were interviewed

^d^ Analysis of mean education did not include missing data of 16 subjects who were not interviewed

^e^ Chemotherapy comprised mainly of anthracycline or taxane-based combinations

^f^ Hormone therapy was based on aromatase inhibitors, anti-estrogens, or combinations

### Completeness and agreement between data sources

Agreement analysis was carried out on the initial study population (119 breast cancer patients) in order to detect all discrepancies between research tools, some of which were liable to disappear following the exertion of inclusion criteria. In the full-scale study, we will perform sensitivity analyses of inclusion criteria in order to investigate the validity and robustness of the findings and conclusions, and therefore, information must be valid for all breast cancer survivors, regardless of compatibility to eligibility criteria. Table 3 displays concordance and completeness results for categorical parameters. Except for cohabitation status, the agreement and predictive values on demographics were significantly very good. In regard to breast cancer characterization and treatment, LHS agreed very well with INCR on breast cancer laterality and type of surgery (*Kappa* ≥ 0.84), but moderately on axillary node dissection. Agreement on adjuvant therapy was calculated for two periods with 2003 set as the cut-off point, for in this year LHS completed the establishment of organized electronic claims. Unlike the low PPVs during the period before 2003, PPVs in years 2003–2010 were above 85 %. The agreement on reported personal medical history with chart review was mostly fair to moderate. The NPVs were almost perfect, translated to low false negatives, whereas the low PPVs indicated high false positive reporting.

**Table 3:** Validity and completeness of patient categorical features across data sources

Characteristic		Data source			Reference Standard	Concordance				Completeness % (proportion)		
		INCR	LHS	QUES		Proportion correct % (*n*)	*Kappa* (*n*, 95 % CI)^a^	PPV (95 % CI)	NPV (95 % CI)	INCR	LHS	QUES
Demographics	Residence	----	✓	✓	QUES	100 (*67*)	----	100 (94.6–100)	----	----	100 (119/119)	100 (67/67)
	Ethnicity	✓	----	✓	QUES	100 (*62*)	1 (*62*)	100 (91.2–100)	100 (85.1–100)	92.4 (110/119)	----	100 (67/67)
	Cohabitation status	----	✓	✓	QUES	60.9 (*46*)	0.55 (*46*, 0.33–0.76)	71.1 (55.2–83)	12.5 (2.2–47.1)	----	73.1 (87/119)	100 (67/67)
	Mortality	✓	✓	----	INCR	100 (*119*)	1 (*119*)	100 (85.1–100)	100 (96.2–100)	100 (119/119)	100 (119/119)	----
Breast cancer characterization and treatment	Laterality	✓	✓	----	INCR	97.8 (*89*)	0.95 (*89*, 0.89–1)	97.3 (86.2–99.5)	98.1 (89.9–99.7)	96.6 (115/119)	75.6 (90/119)	----
	Surgery	✓	✓	----	INCR	100 (*97*)	----	100 (96.2–100)	----	81.5 (97/119)	97.5 (116/119)	----
	Breast conserving surgery	✓	✓	----	INCR	93.8 (*97*)	0.86 (*97*, 0.75–0.97)	98.4 (91.5–99.7)	85.3 (69.9–93.6)	81.5 (97/119)	94.1 (112/119)	----
	Mastectomy	✓	✓	----	INCR	92.8 (*97*)	0.84 (97, 0.72–0.95)	91 (81.8–95.8)	96.7 (83.3–99.4)	81.5 (97/119)	94.1 (112/119)	----
	ALN dissection	✓	✓	----	INCR	79.4 (*97*)	0.50 (*97*, 0.33–0.67)	75.3 (64.9–83.4)	100 (80.6–100)	81.5 (97/119)	92.4 (110/119)	----
	Radiotherapy											
	Before 2003	✓	✓	----	LHS claims	28.6 (*7*)	----	28.6 (8.2-64.1)	----	26.9 (7/26)	34.6 (9/26)	----
	2003-2010	✓	✓	----	LHS claims	87.5 (*8*)	----	87.5 (52.9-97.8)	----	8.6 (8/93)	100 (93/93)	----
	Chemotherapy											
	Before 2003	✓	✓	----	LHS claims	44.4 (9)	----	44.4 (18.9-73.3)	----	34.6 (9/26)	46 (12/26)	----
	2003-2010			----	LHS claims	91.7 (12)	----	91.7 (64.6-98.5)	----	12.9 (12/93)	100 (93/93)	----
	Hormone therapy											
	Before 2003	✓	✓	----	LHS claims	0 (*1*)	----	0 (0–79.4)	----	3.8 (1/26)	65 (17/26)	----
	2003-2010	✓	✓	----	LHS claims	100 (5)	----	100 (56.6-100)	----	5.4 (5/93)	100 (93/93)	----
Personal medical history	Hypertension	----	✓	✓	Medical record	79.1 (*67*)	0.55 (*67*, 0.36-0.74)	51.7 (34.4-68.6)	100 (90.8-100)	----	100 (119/119)	100 (67/67)
	Hyperlipidemia	----	✓	✓	Medical record	73.1 (*67*)	0.46 (67, 0.25-0.66)	59.4 (42.3-74.5)	85.7 (70.6-93.7)	----	100 (119/119)	100 (67/67)
	Renal failure	----	✓	✓	Medical record	100 (*67*)	----	----	100 (94.6-100)	----	100 (119/119)	100 (67/67)
	Rheumatoid arthritis	----	✓	✓	Medical record	97 (*67*)	0.65 (*67*, 0.21-1)	50 (15–85)	100 (94.3-100)	----	100 (119/119)	100 (67/67)
	Diabetes	----	✓	✓	Medical record	97 (*67*)	0.93 (*67*, 0.83-1)	90.5 (71.1-97.4)	100 (92.3-100)	----	100 (119/119)	100 (67/67)
	Osteoporosis	----	✓	✓	Medical record	68.7 (*67*)	0.37 (*67*, 0.14-0.59)	67.9 (49.3-82.1)	69.2 (53.6-81.4)	----	100 (119/119)	100 (67/67)

*Abbreviations*: *INCR* Israel National Cancer Registry, *LHS* Leumit Health Services, *QUES* questionnaire, *CI* confidence interval, *ALN* axillary lymph node

^a^ Agreement analysis was conducted only for symmetric 2-way tables and excluding missing values

**Fig. 3:**
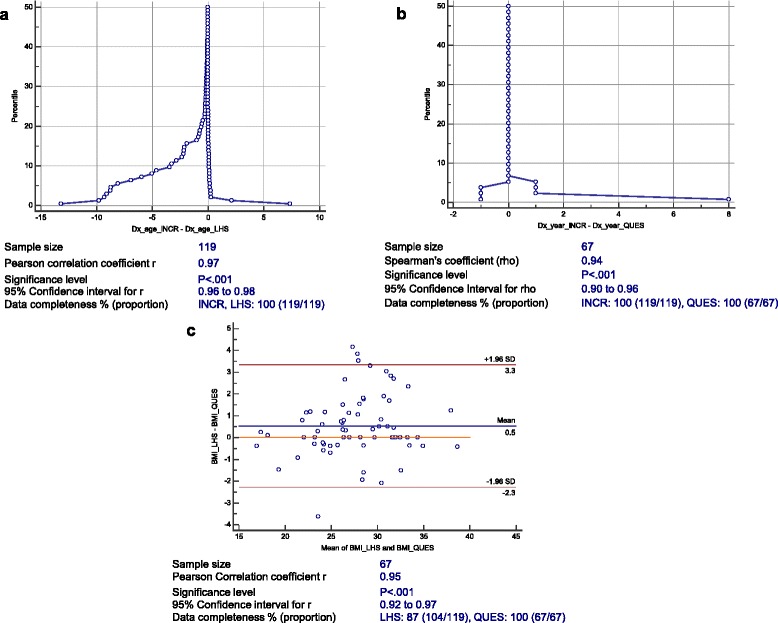
Validity and completeness of patient continuous features across data sources: **a**, Mountain plot analysis of agreement between INCR and LHS on age at breast cancer diagnosis. Median = (−0.04y), percentiles: 2.5th = (−9.2y) 97.5th = 0.2y; **b**, Mountain plot analysis between INCR and questionnaire on breast cancer diagnosis year. Median = 0y, percentiles: 2.5th = (−1y) 97.5th = 1y; **c**, Bland Altman analysis of agreement between LHS and QUES on body mass index (BMI). The mean BMI difference = 0.5 kg/m^2^ (mean weight and height difference, 0.9 kg and 0.6 cm, respectively), and the 95 % limits of agreement from −2.3 kg/m^2^ to 3.3 kg/m^2^ (Measured BMI was up to one year from self-reported BMI)

Agreement between data sources on continuous variables was quantified graphically (Fig. 3). Generally, the data sources were highly correlated (*P < .001*), and the plots demonstrated good agreement between them. Bias between INCR and LHS was nearly zero on age at diagnosis, however the tails were large and skewed to the left (Fig. 3a). In contrast, the 95 % tails of the mountain plot analysis between INCR and the questionnaire on year of diagnosis, an equivalent parameter to age at diagnosis, were only ±1 from the median (Fig. 3b). Bland Altman plot showed a slight bias (0.5 kg/m^2^) and uniform distribution of the variances over most of the measured BMIs. Despite that the self-reported weight or height were within the acceptably ±2.0 kg or ±2.0 cm measured weight or height, respectively, the limits of agreement of BMI were wider than the acceptable well-established limits of ±1.4 kg/m^2^ (Fig. 3c) [23].

LHS claims databases were almost complete, missing information was mainly confined to the period before 2003. INCR database had markedly missing information on adjuvant therapy, whereas data elicited from the questionnaire were 100 % complete (Table 3, Fig. 3).

### Validity of LHS outcome diagnosis coding

LHS administrative databases recorded the diagnosis correctly in most times (≥94 % proportion correct). Agreement with medical records was good for stroke, and very good for all other outcomes. Generally, NPVs were higher than PPVs. Incidence of fractures and most cardiovascular events was low (≤10 %), whereas chronic pain was mildly to severely experienced by more than half of the questionnaire participants (Table 4).

**Table 4:** Validity and completeness of outcomes in LHS administrative databases

Outcome	No. of patients	Reference Standard	Incidence %	Proportion correct %	*Kappa* (95 % CI)	PPV (95 % CI)	NPV (95 % CI)	Completeness % (proportion)
Cardiovascular disease	119	Medical record	20.2	98.3	0.95 (0.88-1)	95.8 (79.8-99.3)	99 (94.3-99.8)	100 (119/119)
Ischemic heart disease	119	Medical record	15.1	98.3	0.93 (0.84-1)	94.4 (74.2-99)	99 (94.6-99.8)	100 (119/119)
Congestive heart failure	119	Medical record	2.5	100	1	100 (43.9-100)	100 (96.8-100)	100 (119/119)
Conduction disorders and cardiac dysrhythmias	119	Medical record	8.4	99.2	0.94 (0.83-1)	100 (70.1-100)	99.1 (95–99.8)	100 (119/119)
Stroke	119	Medical record	7.6	97.5	0.79 (0.55-1)	100 (61–100)	97.4 (92.5-99.1)	100 (119/119)
Fractures	119	Medical record	6.7	98.3	0.85 (0.64-1)	100 (61–100)	98.2 (93.8-99.5)	100 (119/119)
Osteoporosis	119	Medical record	25.2	98.3	0.96 (0.90-1)	93.8 (79.9-98.3)	100 (95.8-100)	100 (119/119)
Diabetes	119	Medical record	15.1	99.2	0.97 (0.91-1)	94.8 (75.4-99.1)	100 (96.3-100)	100 (119/119)
Chronic pain	67	SF-36	68.7^a^	94	0.85 (0.72-0.99)	92 (81.2-96.9)	100 (81.6-100)	100 (67/67)

Abbreviations: PPV, positive predictive value; NPV, negative predictive value; CI, confidence interval; SF-36, 36-item short form

^a^ For chronic pain, the effect measure is prevalence

### Predictors of the accuracy of self-reported personal medical history

In multivariable analysis of factors likely to be associated with agreement between the self-reported medical history of hypertension and chart review, only year of breast cancer diagnosis was significant (*P = .020*). Subjects were more likely to correctly report hypertension the more recently in time that their breast cancer had been diagnosed (OR, 1.61; 95 % CI, 1.08–2.41). Family history of cardiovascular disease predicted correct reporting of hyperlipidemia (OR, 5.12; 95 % CI, 1.21–21.65; *P = .026*). Increasing levels of BMI were associated with better reporting as well and almost reached statistical significance (OR, 1.21; 95 % CI, 0.99–1.48; *P = .057*). Correct reporting of osteoporosis was significantly associated with ethnic disparities. Jewish women were more likely to accurately report diagnosis of osteoporosis than Arab women (OR, 25.30; 95 % CI, 1.48–433.61; *P = .026*). In contrast, increasing number of offspring (parity) was significantly associated with incorrect responses regarding osteoporosis (OR, 0.60; 95 % CI, 0.38–0.95; *P = .029*).

### Questionnaire pretest and validity

Questionnaire administration to all respondents spread over a two-month period. All the participants who completed the survey found it acceptable, and did not refuse to answer any question, although such choice was provided. Language-wise, there were no problems of understanding the content of questions, although some Arab women needed at times translation, especially of diagnoses. We only revised the question on location of chronic pain, since despite respondents were instructed to select only one answer per question, they frequently pointed to more than one location.

Chronic pain intensity positively correlated with number of pain locations (Spearman correlation coefficient, 0.83; 95 % CI, 0.73–0.89; *P < .001*). In a similar manner but to a lesser extent, employment transition correlated with income instability (Spearman correlation coefficient, 0.7; 95 % CI, 0.56–0.81; *P < .001*).

Table 5 provides the SF-36 subscale reliability scores. Cronbach’s alpha coefficients ranged from 0.9 to 1.0, meeting the acceptable level of scale reliability [24]. Except for subscales of general health and physical functioning, the correlations of items with their scales exceeded 0.5. When comparing mean scores of health-related quality of life with the general adult Hebrew-speaking population [17], our sample population was significantly associated with worse health assessment, expressed in lower scores on all eight subscales.

**Table 5:** Reliability of SF-36 form self-reported data

SF-36 Health Form Subscales	Breast cancer sample population						Israeli Adult Population		T-test Sig.
	Standardized Alpha Cronbach	Number of items	Corrected^a^ item correlation with own subscale		Mean score	SD	Mean score	SD	
			Range	Sig.					
Bodily pain	0.9	2	0.9	<.001	63.7	23.9	71.6	29.8	.01
General health	0.9	5	0.4–0.8	<.001	49.0	31.0	62.9	23.8	<.001
Mental health	0.9	5	0.5–0.7	<.001	54.5	28.7	67.1	21.7	<.001
Physical functioning	0.9	10	0.1–0.8	<.001–0.376	59.8	40.0	77.3	26.6	<.001
Role emotional	1.0	3	1.0	<.001	50.7	50.1	81.1	35.7	<.001
Role physical	1.0	4	0.8–1.0	<.001	49.3	50.1	71.3	40.8	<.001
Social functioning	0.9	2	0.8	<.001	59.5	27.2	81.8	26.8	<.001
Vitality	0.9	4	0.7–0.9	<.001	39.3	25.8	56.9	22.8	<.001

*Abbreviations*: *SF-36*, 36-item short form, *Sig* significance, *SD* standard deviation

^a^ Correction refers to recoding per a scoring key [12]

## Discussion

Agreement among administrative databases and self-report constructed questionnaire ranged from moderate to very good. In general, the study data sources highly agreed on patient demographics, with the exception of cohabitation status for which the majority of inaccuracies were LHS misclassification of divorced/widowed women as being married. This may allude to lack of streamlined update of marital status in LHS databases, but may also reflect the dynamics of marital dissolution and social support deprivation due to illness.

LHS administrative claims databases proved to be accurate in regard to adjuvant therapy as beginning from 2003 onward, and can be readily used for adjuvant care surveillance without extensive validation. The low agreement with INCR on axillary node dissection and the slight disagreement on type of surgery render INCR as the preferable method for capturing surgical treatment information, consistent with findings by Turner and colleagues [25].

There were knowledge gaps among breast cancer survivors concerning their medical history, varying by condition and patient characteristics. In accordance with previous reports [26, 27], agreement with medical records ranged largely from fair to good. However, results must be dealt with caution as *Kappa*, much like predictive values, is affected by prevalence of the finding under consideration [28]. In general, women overreported comorbidities. The association between recency of breast cancer diagnosis and correctly-reported hypertension could be attributed to the ongoing management of treatment-related symptoms and side effects [7], and more frequent physician visits. Routine measurement of blood pressure during these visits is considered a quality care since hypertension is not only a comorbidity but a risk factor for cardiovascular disease, and therefore physicians are continually reminded to raise the patient awareness of their medical condition and to emphasize the benefits of strict adherence to hypertensive medications. Similarly, the positive association between correctly-reported hyperlipidemia and family history of cardiovascular disease or increasing BMI levels may be explained by the fact that women with established risk factors for heart disease are monitored closely for high levels of cholesterol [29]. The accuracy of self-reported osteoporosis was the lowest of all comorbidities; the *Kappa* statistic of 0.37 was comparable to that reported by Stuart and colleagues [30]. Reporting inaccuracy may be attributed to patient misunderstanding of their bone mineral density (BMD) test results [30], often misclassifying osteoporosis with osteopenia [31]. Jewish women were less likely to misclassify osteoporosis than Arab women. Ethnicity could be a surrogate for level of education; Jewish women in our sample were more educated than Arab women (Table 2), and could discern osteopenia from osteoporosis more easily. Alternatively, ethnicity could reflect language barriers that hinder Arab women from understanding the term “osteoporosis”. Parity was associated with reduced ability to correctly report osteoporosis as well. Parity may be linked to prolonged lactation period and inadequate recovery of bone calcium [32]. Parous women who subsequently initiate on calcium supplement intake to replenish their body supply may misperceive their condition as osteoporotic.

Self-reported date of breast cancer diagnosis was not biased and was largely within the 95 % agreement limits with INCR. This accuracy could be attributed to subjective perceived importance of the matter [33]. Self-assessment of chronic pain was also shown to be accurate. This is a finding of paramount importance, since when we compared the proportion of women who reported not having chronic pain with painkillers procurement history retrieved from LHS pharmacy claims (a proxy measure for chronic pain), only 6 % of women reporting not having chronic pain did not consume painkillers, rendering administrative databases as unsuitable for assessment of chronic pain.

Outcome diagnosis abstracted from administrative databases exhibited high concordance with medical records. Therefore, it is warranted to use LHS datasets to abstract information on diseases and to drop relevant questions from the questionnaire, with reservations on breast cancer diagnosis date. Notwithstanding the accurate identification of breast cancer cases in LHS databases as compared to INCR (PPV, 99; 95 % CI, 95.4-99.9), diagnosis dates were occasionally incomparable; often INCR preceding LHS. A reasonable explanation of such discrepancy may be embedded in the coding conundrum of active versus history of cancer [34]. As long as breast cancer patients receive treatment for the condition, they continue to be reported with the malignant neoplasm ICD-9 code 174. Only when the malignancy has been excised, there is no ongoing treatment, and there is no evidence of recurrence, the code is replaced with V10.3, personal history of malignant neoplasm of breast. By reason of the extended period of receiving adjuvant therapy, breast cancer cases identified in LHS registry during 2000 and 2010 as active could lag to an earlier period. Another probable explanation could be attributed to the fact that INCR registers the date of the pathological report as the date of diagnosis while the HMO may use instead the date of treatment initiation. It is, therefore, necessary to compare LHS and INCR breast cancer diagnosis dates before commencing on the full-scale study. An easier approach would be identifying patients directly from INCR then linkage with LHS databases; however, INCR is prohibited from releasing information unless provided with a list of patients and an ethics approval from the care provider.

The questionnaire was demonstrated as approvingly valid. The uniform distribution of BMI variances despite the slight wider limits of agreement than the acceptable, the high internal consistency of the quality-of-life subscales, and the large correlation between self-reported pain intensity and number of pain locations or employment transition and income instability, altogether bestow reported information highly-ranked credibility.

Common sense suggests that the shorter the questionnaire, the more likely a high response rate. Surprisingly, not only 99 % of contacted women responded, but they willingly provided complete information. We conjecture that personal contact greatly increased participation rate. Other strengths of this feasibility study include the two-stage pretest of the survey, the definitive validation algorithms of outcomes using the medical records as the reference standard, exploration of potential factors associated with agreement, and almost complete information in LHS administrative databases, the main data source of the study.

The external generalizability of the study results to all Israeli female survivors of breast cancer might be affected, inasmuch LHS is the smallest of four Israeli not-for-profit HMOs. Despite, as previously reported [14], all HMOs are obliged to insure every applicant regardless of age, gender, health condition, or any other criterion, LHS approach to data collection and management may differ from other HMOs, and thus the validity of administrative information might not be generalizable after all to other care funds. Further research comparing cancer care data quality among Israeli HMOs is, therefore, warranted. On the other hand, it is unlikely that breast cancer patients in LHS differ fundamentally from other breast cancer survivors, and therefore, the study conclusions regarding the validity of self-report may be generalized to all breast cancer survivors.

Another shortcoming of this feasibility study is potential selection bias by limiting the sample to women treated within a single institution. As one of our objectives was to examine heterogeneity of treatment, we needed to isolate effect of inter-institution variance in treatment protocol; therefore, we selected one institution which offered all treatments. Additionally, women recruited were oversampled for Arab minority; this could have affected generalizability of results and agreement inasmuch the likelihood of correct responses was associated with ethnic disparities. Nevertheless and more importantly, the principal intention of this pilot study was to evaluate the impact of language barriers on comprehension capabilities of a Hebrew-written questionnaire, which, in our opinion, outweighs this drawback. Additional study limitations include the small sample size as is common for a feasibility study [35]. Although this sample size was sufficient for providing information about all investigated aspects, it might have lacked statistical power, and significant results could perhaps be corollary of chance, which could explain the broad confidence intervals around the estimates.

Our study may have important implications on health policy and clinical practice. Health policy regulators rely substantially on the data produced by clinical research studies to base their informed decisions and institute cost-effective guidelines that affect clinical practice. Policy decisions, such as resource allocation, reimbursement, prevention, surveillance, implementation of health education and promotion activities or disease management programs, however, must stem from valid and credible information, otherwise they may be biased, inefficacious, and may incur expenses that do not benefit either the public or the health care systems, specifically at times of increasingly economic constraint and cost-containment policies. The high quality and integrity of administrative data demonstrated in this study, diagnoses in particular, ensure that these ready-to-use automated information delivery systems can be relied upon and utilized to evaluate cancer treatment late effects and promote policies of survivorship care. Furthermore, the remarkable accuracy of administrative claims treatment and outcome information gives them an edge over the expensive and time-consuming medical records, and sets them as the preferable data source at the policy level.

## Conclusions

The results of this feasibility study demonstrate that in the absence of a national registry for cancer care, a mixed methodology for data collection provides the most complete information: accessing details of adjuvant therapy, medical history and outcomes from HMO administrative databases, collecting data on initial treatment by linkage with cancer registries, and interviewing patients for the added value of lifestyle behavior and subjective appraisal of quality of life. Such an approach is likely to be more comprehensive than collecting patient features by abstraction from individual medical records. The accurate self-assessment of chronic pain should be of use to other researchers who are considering using self-report questionnaires to determine the impact of cancer treatment on chronic pain and related sequelae. Finally, designing meticulous validation schemes and careful piloting are critical for identifying potential flaws and bias in research instruments before launching on a large-scale study which will require data abstraction and participation of breast cancer patients of much larger dimensions.

## Abbreviations

2-PG: 2-Hour Post Glucose-Load Plasma Glucose
ACEI: Angiotensin Converting Enzyme Inhibitor
ALN: Axillary Lymph Node
ARB: Angiotensin Receptor Blocker
BMD: Bone Mineral Density
BMI: Body Mass Index
Chem: Chemotherapy
CI: Confidence Interval
Cm: Centimeter
CT: Computerized Tomography
EF: Ejection Fraction
FPG: Fasting Plasma Glucose
HbA1c: Hemoglobin A1C
HMO: Health Maintenance Organization
Hormone: Hormone therapy
ICD-9: International Classification of diseases, Ninth Revision
INCR: Israel National Cancer Registry
Kg/m^2^: Kilogram/meter^2^
LHS: Leumit Health Services
Mg/dL: Milligram/Deciliter
MRI: Magnetic Resonance Imaging
NPV: Negative Predictive Value
OR: Odds Ratio
*P*: Pvalue
PET: Positron Emission Tomography
PPV: Positive Predictive Value
QUES: Questionnaire
Rx: Radiotherapy
SD: Standard Deviation
SF-36: 36-Item Short-Form Health Survey
Sig.: Significance
